# Fostering Self-Management of Everyday Memory in Older Adults: A New Intervention Approach

**DOI:** 10.3389/fpsyg.2020.560056

**Published:** 2021-01-07

**Authors:** Christopher Hertzog, Ann Pearman, Emily Lustig, MacKenzie Hughes

**Affiliations:** School of Psychology, Georgia Institute of Technology, Atlanta, GA, United States

**Keywords:** intervention – behavioral, everyday memory, memory training, metacognition, shaping, habits and behaviors

## Abstract

Traditional memory strategy training interventions improve older adults’ performance on tests of episodic memory, but have limited transfer to episodic memory tasks, let alone to everyday memory. We argue that an alternative approach is needed to assist older adults to compensate for age-related cognitive declines and to maintain functional capacity in their own natural ecologies. We outline a set of principles regarding how interventions can successfully train older adults to increase successful goal pursuit to reduce risks of everyday memory failures. We argue that training individuals to use metacognitive self-regulatory strategies to proactively manage formulation and pursuit of daily goals can compensate for age-related cognitive changes and increase the likelihood of goal attainment. We then describe an intervention approach that instantiates these principles in a multi-modal intervention that is unique in its three-phase approach: (1) individualized assessment of an individual’s current approaches to self-regulation; (2) training memory strategies, self-management skills, and new habits of mind in a group training context; and (3) a behavioral shaping period in which individuals receive coaching and feedback on their efforts to use trained procedures to improve everyday cognition. A recently completed study conducted an initial test of the intervention, with highly encouraging results. We advocate further efforts to replicate, extend, and fine-tune this type of intervention. The ultimate goal is to be able to deliver the intervention in a way that increases its potential reach, including to subpopulations of older adults at risk for everyday cognitive impairments.

## Introduction

This paper describes a novel approach to improving everyday memory functioning in older adults, laying out the theoretical rationale for the approach and describing its key features. We start with a potentially controversial and surprising point. Despite a plethora of empirical studies and ample federal grant dollars invested, cognitive training research to date has had little impact on the quality of life of older adults. The thinking and research questions that have dominated scientific studies of cognitive training with older adults – e.g., a focus on mnemonic training benefits for standardized cognitive test performance – have limited and perhaps even stifled alternative approaches with the potential to improve older adults’ everyday cognition. The principal message of this paper is that improving older adults’ cognition requires explicitly training the use of skills and habits that will be effectively employed in the ecologies of people’s daily lives.

### Cognitive Training in Older Populations: State of the Field

It is well known that episodic memory, or memory for past events, declines with normal aging (e.g., Hultsch et al., 1998; Zacks et al., 2000; Healey and Kahana, 2016). Older adults are less likely to remember specific contextual details (e.g., Greene and Naveh-Benjamin, 2020) and are prone to a variety of memory illusions (Devitt and Schacter, 2016), some of which can be repaired by effective encoding strategies (e.g., Hertzog et al., in press) and retrieval strategies such as the distinctiveness heuristic (e.g., Gallo et al., 2006).

It is also well established that subjective memory complaints (SMC) have limited validity for predicting actual memory decline in older adults, being more highly correlated with negative affect and depression (see review by Hertzog and Pearman, 2014) in people free of memory pathologies like Alzheimer’s disease.

Episodic memory decline has been a target for efforts to remediate memory through training (e.g., Shing et al., 2008). An extensive literature conclusively shows that older adults’ cognitive test performance can be improved through cognitive training, including improvements in performance on episodic memory tests by training use of memory strategies. Cognitive interventions typically fall into two broad categories (Karbach and Verhaeghen, 2014; Simons et al., 2016): (1) strategy training (e.g., Rebok et al., 2014), or (2) extensive practice on tasks (often, computerized task modules thought to benefit attentional and cognitive control). Memory strategy training typically produces improvement on trained memory tasks without much generalization (or transfer) to different task contexts, including other memory tests (e.g., Bailey et al., 2014; Rebok et al., 2014).

Extensive task practice also shows practice-related benefits with limited transfer (e.g., Schmiedek et al., 2010). Training benefits accrue when processes are honed that can be directly applied in other environments, such as maintaining a focus of attention and controlling working memory under cognitive load and background distraction (e.g., Fraser and Bherer, 2013; Rolle et al., 2017). Here too, transfer is most likely when trained cognitive processes are directly afforded by the transfer task environment (Lövdén et al., 2010).

It is an open question as to whether either type of cognitive training leads to better cognitive functioning by older adults in everyday life. The ACTIVE study (Rebok et al., 2014) found at 10-year follow-up that their three cognitive training groups (reasoning, memory, and speed of processing) had better self-reported instrumental activities of daily living (IADLs) than a passive (testing-only) control group. No differential effects, however, were seen on everyday problem-solving tests. Training speed of processing via the Useful Field of View (UFoV) Test improves performance on a simulated grocery-shopping task that assesses “timed IADLs” (Edwards et al., 2018). Based on these and other findings, Edwards et al. (2018) argued that the attention and speed of processing training instantiated in the UFoV benefits everyday cognition and complex IADLs that require it (e.g., driving, banking, medication adherence).

There are at least two concerns with inferring transfer of training to everyday cognition from these studies. First, the UFoV training module provides explicit feedback that the onset asynchrony between a briefly presented target display and a backward pattern mask is being reduced by practice. That feedback may persuade participants that the UFoV training truly is “speeding up their brains.” However, stimulus-specific perceptual learning, not improvements in visuospatial processing speed, could account for the UFoV improvements. Nevertheless, the feedback provided during training may increase older adults’ subjective confidence in their speeded visuospatial processing ability – a kind of placebo effect (Simons et al., 2016). Sharpe et al. (2014) argued that expectancies and self-efficacy cannot account for UFoV training benefits; however, the questionnaires they used to support this claim do not assess process-specific confidence in the UFoV task context – the kind of localized measure advocated by self-efficacy theorists (Bandura, 1989).

Second, most of the demonstrated transfer effects in the literature are not actually to everyday cognition in the natural ecology, but rather to tests or tasks designed to simulate it (and even some of those measures did not show transfer in the ACTIVE study). Typically, tests designed to simulate everyday cognition correlate highly with tests of psychometric intelligence (e.g., Allaire and Marsiske, 1999), possibly implying that performance on these tasks is driven by individuals’ cognitive resources needed to comprehend test instructions and to create strategies spontaneously to meet task demands (Stine-Morrow et al., 2006).

The grocery shopping simulation task used by Edwards et al. (2018) has good face validity, but its predictive validity for actual grocery shopping behaviors in everyday life is unknown. For one thing, real-world shopping would not be evaluated in terms of time to make desired purchases. However, a series of studies reported that UFoV training has beneficial effects on real-world driving behaviors and outcomes like driving cessation or accident rates (e.g., Ross et al., 2017), which would directly address the concern about transfer effects to behaviors in real-world contexts (but see Lintern and Boot, 2019).

Clearly there are still open questions surrounding the evidence for transfer of cognitive training experiences to everyday cognition. Be that as it may, there is little evidence at present that either strategy-oriented or practice-oriented interventions targeting memory processes actually improve older adults’ everyday remembering. We claim that the goal of improving older adults’ everyday cognition may be better realized if the training regimen is based on an entirely different approach that is beginning to emerge in this area – one with different behavioral targets and different training methods that emphasize training for and evaluating everyday cognitive behavior.

### Stated Principles for an Alternative Approach to Training Everyday Cognition

Our intervention approach is based on several principles, laid out in detail in this section. A key feature is that our approach does not focus exclusively on episodic memory, but instead broadly trains everyday memory, which emphasizes prospective memory, use of memory supportive techniques (like external memory aids), and self-regulatory behaviors.

#### People Are Creatures of Habit That Are Often Error-Prone

Everyday cognition is typically grounded in idiosyncratic daily routines and habits (Wood and Rünger, 2016). People evolve styles of living early in adulthood that encompass a set of habitual procedures for accomplishing daily task requirements (Phillips et al., 2016). We have preferred ways of preparing meals and accomplishing other activities of daily living, including instrumental activities such as shopping, banking, and household maintenance.

One can regard daily habits and routines through the same lens used to evaluate problem solving heuristics (Kahneman, 2011). Routines and habits evolve in part because they are effective, but also because they minimize cognitive load. Gigerenzer et al. (1999) argued that fast and frugal problem-solving heuristics evolve in natural ecologies because they are effective while also minimizing the need for effortful cognitive control. Older adults, like younger adults, often rely on heuristics rather than analytic logic (Peters et al., 2007), probably benefiting from a lifetime of experience in acquiring practical experience and adapting routines and habits to meet ecological contingencies (Mata et al., 2012).

However, there is reason to believe that everyday habits and routines are vulnerable to error (Cavanaugh et al., 1983; Vestergren and Nilsson, 2011). In particular, when atypical circumstances or disruptions of routine behaviors occur or when a situation requires multitasking, older adults are more likely to forget to complete intended actions (Adams-Price and Gonzalez, 2005; Weakley and Schmitter-Edgecombe, 2019). Risks for everyday cognitive failures may be accentuated by age-related cognitive declines, given inertial resistance to change or adaptation for needed compensation for age-related changes (Bäckman, 1989; Farias et al., 2018). Hertzog et al. (2019) recently observed some older adults reporting reliance on incidental memory – assuming that if they merely attend to information, they will remember it when needed. Although attention is indeed a critical prerequisite for forming new memories (e.g., Craik, 2002), incidental memory declines over the adult life course (Schneider and Pressley, 1997). A habitual reliance on incidental remembering, formed in early adulthood and fostered by the normatively accurate belief that “important things will be remembered,” is likely to increase the risk of everyday memory failures as adults grow older (Hertzog et al., 2019).

#### People Are Often Reactive, Not Proactive

Conscientious individuals often plan their future actions. However, many adults have evolved a style of behaving that is essentially stimulus driven. They habitually react and adjust to situational demands, maintaining ‘cognitive economy’ but with a cost. Experimental studies indicate that older adults prefer to be passive and reactive when responding to discriminative cues, more so than younger adults, although this tendency can be reduced by training or monetary incentives (Yee and Braver, 2018). We speculate that this pattern may reflect an experience-derived bias toward responding to problems on the basis of using a heuristic of recognizing cues as indicative of past experiences. Recognition then activates familiar problem-solving strategies (Pachur et al., 2009). A reactive everyday problem solving mode may also be an outcome of a motivation to avoid allocation of cognitive resources (Hess, 2014).

Older adults’ use of successful everyday problem-solving strategies is often driven by experiential factors, including a lifetime of observing which strategies work and which do not in various situations (Blanchard-Fields, 2009). Experience may promote a reactive problem-solving style in everyday life; however, this style may be more error-prone than a proactive form of problem-solving that anticipates future demands and how to address them. In particular, a proactive approach toward everyday prospective memory, involving the use of implementation intentions, is a highly effective means of insuring successful future action (Hering et al., 2014; Waldum et al., 2016).

#### Using External Aids Does Not Equate to Using Them Effectively

Habits and routines also evolve around how people use external memory aids, such as calendars, lists, bulletin boards, and appointment books. Older adults report frequent use and reliance on external aids (e.g., Cavanaugh et al., 1983; Hultsch et al., 1987; Gilewski et al., 1990). Furthermore, older adults claim they have control over everyday remembering through the use of external aids (Hertzog et al., 2010).

Researchers have typically not investigated how people actually make use of these external memory aids. Surprisingly, the available metamemory questionnaires assessing everyday memory strategies (e.g., Dixon et al., 1988; Gilewski et al., 1990; Dixon and de Frias, 2007) do not cover subjective effectiveness, method of use, or whether external aid usage contributes to successful memory support. Instead, the implicit assumption seems to be that if people report using external aids, they must be doing so in a way that is effective.

Use of an external aid does not protect against everyday memory errors when the habits and routines in which aids are embedded are prone to misuse. For example, making a grocery list may be accomplished in a way that identifies all of the desired goods for a shopping trip. But even if a list is constructed, one may fail to use it effectively to remember to make all the requisite purchases. One might leave the list at home or in the car. One might not consult the list while shopping, check off items as they are collected, and monitor the list for additional needed goods before checking out. When people were explicitly interviewed about using lists, they generally reported a procedure for creating lists but said little about procedures for using lists effectively (Hertzog et al., 2019). We believe that training individuals to evaluate and optimize their methods for using external aids will have salutary effects on their everyday remembering.

#### Keep It Simple

One feature of memory strategy training studies is that they often teach complex mnemonics that are difficult to learn. There can be good reasons for such approaches in a scientific evaluation of aging and memory. Kliegl et al. (1989) used the method of loci to study older adults’ ability to use interactive imagery to learn serial lists of words, finding increased age differences in memory after training. This approach was argued to ‘test the limits’ on older adults’ cognitive plasticity, finding it to be constrained by age related declines in episodic memory (e.g., Shing et al., 2008).

The ACTIVE project’s memory training module trained older adults on several mnemonic strategies, including the method of loci. Older adults showed immediate posttest improvements in serial memory – which the method of loci targets – but the data on long-term strategy use by the memory-trained ACTIVE cohort was far from impressive. Gross et al. (2014) found that just 25% of their trained participants had patterns of subsequent follow-up test behavior consistent with use of the method of loci on a multiple-trial serial memory task. This result suggests a majority of their participants did not maintain use of the method on the targeted memory task after training. It stands to reason that even fewer of their participants used it in everyday life.

A similar concern exists for another complex mnemonic often recommended for learning new names: using bizarre imagery to create an association of a person’s facial features to their name (McDaniel and Einstein, 1986). Attending a social event may require learning new names, a task older adults report to be a major memory challenge (e.g., Cohen and Faulkner, 1986). However, using bizarre imagery to learn new names is difficult to master, time-consuming to implement, and hence not always successful in actual social situations. It is not necessarily an effective strategy for older adults’ face-name learning (e.g., Poon and Walsh-Sweeney, 1981).

Successful use of memory encoding strategies is a function of the affordance of a given task context to a candidate strategy (e.g., Dunlosky et al., 2003; Bottiroli et al., 2010; Lemaire, 2010; Bailey et al., 2014). We argue that both the method of loci and the bizarre imagery approach are ill-suited to everyday use. It is difficult and time-consuming to form bizarre images, excessively so in an actual social situation as one also engages in the primary goal of social interaction. Regarding the method of loci, how often are people faced with the need to learn a series of words in serial order, and if they were, would they be likely to use the method of loci for this purpose? Perhaps the difficulty in using these mnemonics accounts for why scientists who study memory do not often use them (Park et al., 1990).

In contrast, there are memory encoding and retrieval strategies that are relatively easy to learn and implement – strategies that are both simple and effective. Our intervention approach seeks to train this kind of strategy in lieu of more complex mnemonics.

Spaced retrieval is a prototype of a simple but effective strategy for everyday remembering. It is a testing-based strategy that has been shown to be highly effective for improving older adults’ remembering, including learning new names (Brédart, 2019). Spaced retrieval involves repeated retrieval of information, linked to either explicit or implicit cues. Our intervention trains people to use spaced retrieval to learn new names, encouraging its use in actual social situations (see also Wiegand et al., 2013). Other simple but effective strategies are also part of our training approach (see below). We argue that training these kinds of strategies is more likely to lead to their use in everyday life, compared to complex mnemonics.

#### Build a Skill Set, Then Train Its Use

We argue that an effective training regimen must train multiple skills directly relevant to everyday use. We use the metaphor of filling a toolbox with tools that can be handy for different purposes. One wants a strategic repertoire that contains different possible strategies (Lemaire, 2010). One advantage of this metaphor is that it encourages people to think about how different strategies are more or less useful given different everyday memory goals. A critical aspect of training then, is not the isolated training of specific strategies but also an emphasis on matching strategies to contextual demands. One would not seek to pound in a nail with a wrench; one would not seek to support remembering to perform an action by using relational strategies to encode a list of words. In essence everyday memory training must focus on both filling a toolbox and teaching people to select tools appropriate to their particular goals and circumstances.

#### Explicitly Train for Contextual Adaptation

Variation in situations and opportunities for realizing everyday goals creates constraints on whether people can effectively use trained strategies. Our view on the limited transfer of trained mnemonics is that training a strategy in a particular task context does not address how that strategy could be adapted or generalized to different, alternative situations. Studies that explicitly coach adapting trained strategies to different task contexts have achieved broader strategy generalization (e.g., Bottiroli et al., 2013), even to simulated everyday cognition (Bottiroli et al., 2017). Explicit discussion of how a trained strategy could be modified or adapted to a different task led to better transfer, including tasks that had not been explicitly discussed during adaptation training. This approach can be characterized as seeking to create active learners rather than passive recipients of trained procedures (Dunlosky et al., 2011).

Our everyday memory training approach builds on the concept of creating active learners. When training a specific strategy, like spaced retrieval for face-name learning, we explicitly encourage people to think about how they could use this technique in other situations for additional purposes, and we provide some explicit coaching in how to do so.

#### Mindful Self-Regulation Can Circumvent Many Avoidable Everyday Memory Failures

Everyday cognition can benefit from using self-management skills that promote effective everyday functioning. Our theoretical approach is grounded in a self-regulatory metacognitive perspective on how people can achieve control over cognitive demands by using techniques to regulate their behavior in contexts (Hertzog and Dunlosky, 2011). We train self-regulation as a behavioral approach that can override counterproductive and ineffective habits common in everyday life (Dunlosky et al., 2011).

We assume that people must be sensitive to cognitive demands in their day-to-day ecology and must be trained to use specific skills or techniques to address those demands. The core principle in our “learner-based approach” is that people without frank physical and cognitive disability can manage themselves and can align their behaviors to address environmental demands despite challenges created by the effects of normal aging. A recurring theme is the importance of being aware of situations and their likely cognitive demands.

Consider the example of learning new names. Individuals may have an appropriate technique for learning a new name – like spaced retrieval – in their toolbox, but that does not insure they will use it. The primary goal of socializing may inhibit an explicit effort to learn a new name upon an introduction. Conversely, activating the goal of new-name learning in the moment can lead to successful use of the strategy.

In effect, less effective routines and patterns of habitual behavior need to be supplemented, if not supplanted, by creating new habits of mind that manage everyday cognition. Moreover, people often need to think about adaptive adjustment of strategies depending on circumstances that may necessitate unusual remedies when everyday goal pursuit is frustrated or challenged. For instance, if on a given day a person forgets their appointment book at home (or their smartphone with its calendar app), what is the backup strategy for remembering those appointments? Anticipating the need for alternative approaches – backup plans as it were – is potentially part and parcel of a successful proactive self-regulatory strategy.

#### Negative Beliefs Often Prevent People From Realizing Training Benefits

Older adults believe their memory to have declined from young adulthood and they often attribute such changes to uncontrollable factors, such as genetics and biological aging (Lachman et al., 1992; Lineweaver and Hertzog, 1998). Furthermore, older adults internalize societal stereotypes of age-related memory decline (Hummert, 2011). These beliefs appear to contribute to SMCs in older adults, reducing the validity of SMCs for predicting actual memory decline (e.g., Hertzog et al., 2018; but see Hohman et al., 2011). Often, multi-modal memory strategy intervention programs (e.g., Stigsdotter-Neely and Bäckman, 1989; West et al., 2008; Wiegand et al., 2013) include belief restructuring components. This approach typically challenges prevailing negative views about aging and memory, seeking to supplant them with a more adaptive set of beliefs regarding the amenability of everyday remembering to the effective use of cognitive skills (Elliott and Lachman, 1989). To the extent that an older adult believes there is little they can do to compensate for age-related memory change, they may either (1) expend little effort to learn new strategies for memory self-management, or (2) be vulnerable to abandoning strategy use if confronted with initial difficulties in implementing strategies. Conversely, fostering memory self-efficacy and perceived control encourages persistence in learning new memory-supportive skills (Bandura, 1989; Berry and West, 1993).

Belief restructuring is often successful in altering negative beliefs about aging and memory. However, these effects are fragile and often regress to baseline (e.g., Lachman et al., 1992). We conjecture that after belief restructuring, individuals experience subsequent everyday memory failures that reactivate memory complaints and concerns causing regression to former beliefs. Older adults who fail to achieve unrealistic memory task performance goals experience a loss of confidence and motivation that adversely affects later performance (West et al., 2003). However, if (1) belief restructuring improves a sense of control over memory, accompanied by an intervention that is successful in teaching new everyday memory strategies, and (2) individuals experience the benefit of using those strategies, possibly with explicit feedback and reinforcement for success, then it may be possible to maintain a belief in the potential for compensation to have a lasting beneficial impact.

#### New Habits Are Hard to Learn, and Require Extensive Experience and Feedback

The literature on habit change in a variety of domains, including new health-promoting habits like diet or exercise, shows that it is difficult to form new habits even when individuals are motivated to change (Wood and Rünger, 2016). Our intervention aims to introduce new habits of mind, in terms of everyday self-regulatory strategies, that are also likely to be difficult to inculcate.

Habits can be changed with sustained practice, supported by corrective feedback and positive reinforcement for behavioral change (Wood and Rünger, 2016). For this reason, our intervention approach combines a standard training regimen with a behavioral shaping period following training that provides interaction with project staff to review reports on how people implemented trained strategies and whether strategy use resulted in memory successes or failures.

#### One Size Does Not Fit All

It is standard practice to administer cognitive training interventions in a uniform manner, with trainers strictly conforming to an established protocol. Such approaches derive from the general design principle that there should be minimal deviation from protocol and that the influence of unwanted sources of error variance should be minimized.

However, the goal of standardized program delivery should not trump the goal of tailoring interventions to match the needs and requirements of particular individuals. Here we take a page from work in occupational therapy and cognitive rehabilitation, where an initial assessment of an individual’s issues and capabilities precedes creation of a treatment plan that is tailored to the individual, in terms of current status and goals for desired function (e.g., Abreu and Toglia, 1987). This is a necessary feature of clinically relevant practical interventions.

Imagine attempting to train use of a smartphone calendar application program with an older adult who owns a smartphone but lacks any familiarity with advanced options other than placing a phone call. Although the app could in principle provide major benefits in terms of tracking and keeping appointments, training the novice older adult user who may have negative beliefs about using advanced technology (Czaja et al., 2006) to do so might prove to be challenging, particularly if that individual already keeps a physical paper appointment book and strongly prefers using it. We claim that optimizing everyday cognitive self-regulation is often best achieved, especially in a relatively short intervention, by adjusting the intervention content to modify and enhance an individual’s existing routines and habits. Our everyday intervention uses a standard approach to introducing new everyday strategies, but it adapts external aid training to work with people’s preferred approaches to managing everyday life. Optimizing peoples’ current behaviors may provide a better pathway to improvement than training use of unfamiliar and perhaps less-desired aids.

#### Assessing Everyday Cognitive Success Requires Measuring Real World Behaviors and Outcomes

Some multi-modal memory training protocols for older adults (e.g., Troyer et al., 2008; West et al., 2008; Wiegand et al., 2013; Cohen-Mansfield et al., 2015) already cover techniques we have emphasized as important, such as spaced retrieval and external aids. However, these techniques have not been the principal focus of the intervention program. Furthermore, intervention protocols that include techniques like spaced retrieval do not directly measure whether people actually use these techniques before, during, or after training, whether they use them well, and what benefit they gain by doing so. Typically, outcomes relevant to everyday memory emphasize self-reports of everyday strategy use (e.g., Troyer, 2001; Troyer et al., 2008).

We advocate the use of daily diary questions and other methods to assess use of trained strategies in everyday life. These measures can be used in short-term within-person assessments to evaluate reported everyday memory successes and failures (e.g., Mogle et al., 2017). In our view, such methods are superior to tests that simulate everyday memory and cognition for evaluating people’s actual behaviors, even though diary responses are subject to self-report distortions and biases. Simulations of everyday cognitive tasks can certainly be informative. Ironically, however, there are very few studies that have validated these simulations against directly measured everyday memory (but see Beaver and Schmitter-Edgecombe, 2017).

One should also not assume that questionnaire measures asking for self-ratings on aspects of everyday memory have good predictive validity for actual behaviors. Hertzog et al. (2000) demonstrated that questionnaire-assessed memory complaints had nil to weak correlations with medication adherence as measured by electronic records of people opening pill bottles at home. However, an interview-based self-report of difficulties remembering to take each medication individuals were taking, collected before medication adherence was assessed, prospectively predicted later adherence. Inferences about everyday memory failures based on specific behavioral self-reports can be valid and have superior predictive validity for everyday cognition.

## The Everyday Memory and Metacognitive Intervention (EMMI) Approach

Our everyday memory intervention approach focuses on shaping the effective management of individuals’ memory-demanding real-life. We have benefited from other interventions that, while not primarily focused on everyday memory outcomes, have successfully implemented some of the approaches we have integrated into this approach (e.g., West et al., 2008; Wiegand et al., 2013; Cohen-Mansfield et al., 2015; Kinsella et al., 2018). We provide some additional details on prior approaches in the context of introducing the components of EMMI.

The EMMI has three phases: (1) an extensive face-to-face semi-structured interview to establish individuals’ current behavior patterns; (2) training modules, working with small groups of older adults; and (3) an intensive shaping period in which individuals file daily self-reports on everyday memory events and those data are used to coach changes to those trained behaviors. The Group Learning Experiences (GLEs) of Phase 2 specifically targets and trains procedures for addressing common instrumental tasks, including remembering appointments, remembering to execute intended plans (prospective memory), learning new names in social contexts, and planning and carrying out daily tasks and errands (see Table 1).

**TABLE 1:** Summary of Group Learning Experience (GLE).

Topic	Content
**Day 1**	
Beliefs about memory	Provide overview of how memory beliefs might influence people’s self-evaluations as well as inhibit performance Focus on restructuring maladaptive memory beliefs as a way of challenging people’s view of what they have control over with their memory
Intentional encoding	Define intentional encoding by comparing it to incidental encoding Encourage participants to think about ways they do/don’t intentionally encode new information Discuss importance of intentionality in encoding new information
Mindfulness	Introduce concept of mindfulness and how it might relate to everyday memory practices and habits of mind
Active noticing	Teach the mindful technique of being aware of one’s surroundings and experiences Practice activities based on West et al. (2008)
Spaced retrieval	Explanation of spaced retrieval and its benefits Practice activity learning the names of people in class
Homework	• Memory belief restructuring in daily life • New name learning for next class
**Day 2**	
Homework review	Review homework assignments Practice class and research team names
Self-testing	Define self-testing and provide some basic research on its benefits Explain how self-testing relates to both intentional encoding and spaced retrieval Generate ideas about how to use it in daily life Practice activity with to-do list
Habits and routines	Define habits and routines Discuss the pros and cons of habits and routines Help identify personal habits/routines and evaluate how they help and hinder effective everyday functioning with individual breakout sessions
Implementation intentions	Define implementation intentions for prospective memory goals Demonstrate an example implementation intention action goal Practice activity with to-do list
Stop, Think, Plan, Act (STPA)	Introduce STPA and explain how it can be used in daily life to enhance memory Identify possible personal uses for STPA
Homework	• Attend to and identify personal habits and routines • Practice using STPA in daily life • Self-testing practice
**Day 3**	
Homework review	Review homework assignments Practice class and research team names
Review of STPA	Review STPA from Day 2
External aids	Discuss types of external aids Identify currently used aids for each person Discuss “optimal” external aid use with a focus on medication management and calendar use
Mindfulness	Review mindfulness from Day 1 Explain how it might be integrated into everyday memory actions Practice activities – diaphragmatic breathing and 5 sense awareness
Homework assignments	• Goal setting • Explain daily diaries • Explain shaping period

### Phase 1: The Intake Interview

The face-to-face semi-structured interview is specifically designed to discover how individuals manage their lives, how they use habits and routines to support everyday cognitive demands, and how they use strategies and external aids to serve everyday goal pursuits. Follow-up questions to initial probes elicit rich information about possible points of risk for everyday memory failures. Interview content is reviewed with the express goal of discussing with participants ways in which their everyday self-management approaches could be improved. For instance, Hertzog et al. (2019) identified individuals who used multiple calendars without a consistent strategy for managing them. Based on that information, EMMI for these individuals would recommend integrating calendars and would train how to approach filling and consulting calendars on a daily basis.

The interview also allows us to assess if individuals are overconfident in their approach to everyday memory. Hertzog et al. (2019) frequently observed individuals relying on the assumption that important information will be remembered, even though important events like refilling prescriptions are infrequent actions and often only cued when medicine containers are low or even empty.

We use these interviews to identify ways in which individuals can be coached to improve use of strategies and external aids and bring ideas about change into individually tailored components of the intervention. The information garnered from these interviews is used in the individual breakout sessions in the group experiences as well as during the shaping procedure. Use of this information helps us tailor the experiences of each participant and is unique in cognitive training paradigms.

### Phase 2: Group Learning Experience

Phase 2 is focused on training memory skills, habits of mind, and external aid use. Table 1 provides an overview of the Phase 2 GLE components which are described in more detail below. The training uses PowerPoint-mediated presentations to small groups of adults with frequent opportunities for group discussion of ideas, experiences, and approaches. The training also includes activities and break-out sessions where individuals can brainstorm with one another and research staff ways to optimize their memory-supportive behaviors. Individuals are assigned homework exercises to complete between sessions that implement and illustrate trained procedures; they are then encouraged to share with the group their experiences using these new techniques. The training is done in a supportive manner and encourages people to regard trained techniques as tools that can be mastered with practice.

#### Component 1. Belief Restructuring

As already reviewed, belief restructuring has been a featured component of multimodal memory strategy training programs for decades. Our belief restructuring component addresses maladaptive beliefs typically targeted in training studies, including the belief that memory decline is inevitable and unavoidable. We focus on supplanting such beliefs with the view that age changes do occur but can be compensated for by using specific techniques.

However, belief restructuring is also needed regarding beliefs that might otherwise undercut adherence to the intervention, including: (1) belief in the efficacy of incidental encoding and retrieval strategies in old age, (2) creating a new belief that proactive self-management is a key means to achieve everyday goals, (3) doubts about one’s capability of learning simple memory skills for addressing problems (e.g., spaced retrieval for learning new names), and (4) inertial resistance to changing current behaviors (Hertzog, 2008). One concern we emphasize is that learning new skills takes time and tolerance for a level of initial failure. We emphasize that the intervention cannot prevent all everyday memory issues, but that it can increase the likelihood of achieving everyday goals and reduce the likelihood of potentially costly errors.

#### Component 2. The Metacognitive Toolbox

The toolbox can include in principle a wide variety of different skills or strategies for supporting everyday memory. The intervention as we now implement it focuses on establishing four specific skills that are simple, easily learned, generically helpful, but often unknown to the public: (1) active noticing (mindful attention), (2) spaced retrieval, (3) self-testing, and (4) implementation intentions to promote successful prospective memory. *Active noticing* capitalizes on the demonstrated benefits of deliberate attending to information for later remembering (see West et al., 2008). The idea is that intentionally attending to information and, if appropriate, attaching meaning to it is a form of intentional, ‘deep’ encoding (Schneider and Pressley, 1997; Craik, 2002) that has profound benefits for subsequent memory. We also emphasize that active noticing can avoid mindless errors. For instance, deliberately encoding where one has parked a car before entering a shopping mall increases the likelihood of locating the car upon returning to the parking lot. We train active noticing through observing information in pictures, encouraging visualization of picture content.

*Spaced retrieval* strengthens accessibility of memorized information committed to memory. It involves self-initiated remembering of target information, optimally with repetition on an expanded schedule of delays between retrieval attempts (Camp, 2006). Older adults typically have not heard of the technique, are surprised that it is effective, and hence have not previously considered using it to learn new information. Spaced retrieval is highly effective for shaping remembering even in cognitively impaired older adults (e.g., Troyer, 2001; Bourgeois et al., 2003; Camp, 2006; Ozgis et al., 2009).

As noted earlier, spaced retrieval is particularly effective for learning new names in social situations. We emphasize three aspects to implementing spaced retrieval of proper names in actual social situations: (1) remember the goal of learning a new name; (2) use active noticing to attend to the name carefully, insuring it ‘enters’ the memory system; (3) repeat the name with spaced retrieval, preferably on an expanding schedule. One first encodes the name, repeating it silently a few times. After a short delay, one explicitly retrieves the name and does so again with some delay between retrieval attempts. In a social situation, this can be done by using a person’s name in conversation with them or introducing them to another person.

Spaced retrieval has a profound benefit for remembering the name later, especially if the retrievals are made over an extended time span (say, several minutes or even hours). It is also easily generalized to other learning contexts. We train new name learning with spaced retrieval early in the intervention because experiencing its success is highly reinforcing and underscores that older adults can learn new names if given the right tools.

*Self-testing* is a metacognitive procedure for enhancing new learning shown to be effective for older adults (e.g., Dunlosky et al., 2003). People test themselves with cues (as with foreign language vocabulary flash cards) to check whether they can actually retrieve information they have been studying. Successful retrieval strengthens memory accessibility. Anything that cannot be recalled is selectively targeted for further study. In our intervention, self-testing is introduced as a way of memorizing a daily to-do list that can be a backup should one misplace or forget to bring along a to-do list when running errands. Older adults can be trained to use self-testing in a single training session or at home with a training manual, producing effects on memory tasks that exceed benefits from mnemonic training (Dunlosky et al., 2003).

Any plan for future action can be translated into an *implementation intention* – a specific plan for how to act (e.g., Gilbert et al., 2009). The technique involves formulating a concrete plan for how to accomplish an action goal at the proper time and place, with an emphasis on visualizing the enactment to benefit from that imagery. Generating implementation intentions increases the likelihood that older individuals will retrieve action intentions at the time and place they are needed to act (e.g., remembering to check blood glucose; Liu and Park, 2004; Altgassen et al., 2014). Interventions that improve prospective memory should include implementation intention training as a feature (see Hering et al., 2014; Waldum et al., 2016). A recent study reinforces the value of both rehearsing implementation intentions and using visualization of the actions as a mediator for context-intention associations that promotes spontaneous retrieval of the intention (Henry et al., 2020). Both of these aspects are emphasized as part of our approach to training prospective action.

#### Component 3. External Aid Training

The third component of our training program involves training the effective use of external memory aids (e.g., Bourgeois et al., 2003; Wiegand et al., 2013). We specifically target medication adherence as one form of everyday memory applicable to virtually all older adults that is often supported by external aids. We also target remembering appointments, making [and using] grocery lists, and scheduling instrumental activities of daily living. We also ask individuals to identify any additional areas where they believe they are experiencing difficulties in memory they would like to address. The goal is generating a set of personalized recommendations for each participant for how to optimize their external aid use.

#### Component 4. Self-Regulatory Habits of Mind

We also encourage people to use self-management habits for effective remembering in everyday life (Dunlosky et al., 2011). We assume that trained strategies (i.e., the toolbox) must be incorporated into new habits of mind that explicitly consider memory demands of everyday life situations and how to best address them. Even if one knows that spaced retrieval helps learn new names, one must remember to use it when encountering new people. We encourage proactive approaches to planning one’s day to anticipate memory-related needs and demands which includes reviewing that plan at the start of each day (or even the night before). Our interview study (Hertzog et al., 2019) suggested this approach is not commonly adopted by older adults, perhaps because they believe daily routines obviate the need for such planning. These habits of mind are integrated into all aspects of the GLE protocol.

#### Component 5. Mindful Self-Regulation

Our intervention also trains older adults to override (as needed) routine habits of behavior which are endemic to normal living but which can lead to self-defeating lapses in remembering. We emphasize a mindful self-regulatory approach labeled STOP, THINK, PLAN, ACT [STPA] that trains people to stop and think about likely memory demands before they enter new situations. We encourage individuals to reflect in the moment, asking: “What am I doing? What comes next? What will be the demands?” This should be done before embarking on a change of location, such as a trip to run errands, but it can be generically useful during the course of a day. Implementing a self-regulatory focus is fostered by proactive goal-setting and self-testing to memorize a to-do list. Being able to explicitly recall upon reflection impending tasks enables a review of whether additional actions are needed, such as fetching all required materials before leaving to do errands. The efficacy of such an approach is supported by a previous study showing that randomly texting “STOP” to brain-injured adults several times a day (with instructions to reflect on intended actions) materially improved everyday prospective memory (Fish et al., 2007).

We encourage people to cease automated behaving (often as part of a routine or habit pattern that people colloquially refer to as “being on auto-pilot”). Instead, we suggest that people learn a new habit of using STPA just before initiating a new action or set of actions by pausing, thinking about the situation, their goals, and next steps toward achieving those goals. The aim is to replace routinized behavior with a conscious, explicit, self-regulatory focus on managing life and its cognitive demands. For instance, when going to the store, stop and check whether the grocery list and method of payment are in one’s possession.

### Phase 3: Behavioral Shaping

Shaping new behavioral habits, including habits of mind, is notoriously difficult and requires cycles of behavior, feedback, and adjustment to ingrain the new habit (Lally and Gardner, 2013; Wood and Rünger, 2016). One learns from mistakes, and mistakes tend to be plentiful during the process of replacing a familiar habit with a new, more mindful one. Our intervention approach follows the GLE training just described with frequent interactions with trainees over several weeks to help them monitor and adjust self-regulatory behaviors. We review participants’ successes, failures, and obstacles to implementing the self-regulatory approach, using frequent telephone calls to review memory successes and failures they have recorded in a daily diary. We provide positive reinforcement for effective use and help them review how to adapt behavior in the case of ineffective use. We also encourage individuals to set and attempt memory-related goals between telephone calls to promote continued and new use of the trained memory strategies. This shaping process is unique in cognitive training studies and provides our participants with the extra support they need to incorporate the techniques into their daily lives.

### Measuring Everyday Memory Outcomes

In order to assess everyday memory outcomes after the intervention, we consider it essential to measure actual success and failure outcomes that individuals experience (Schmitter-Edgecombe et al., 2020). Our first study adapted a self-report procedure from Mogle et al. (2017), embedded in an online nightly diary format, that includes a checklist of everyday memory problems an individual may have encountered. We supplemented this method by also asking people to specifically report memory successes and memory failures (labeled “memory blips” to avoid the negative connotations of “failure”).

To assess intervention engagement, we also asked individuals to report what techniques they used each day to help support everyday remembering by completing a strategy checklist (e.g., using an appointment book or calendar, using a smartphone alarm).

It is also highly desirable to collect behavioral indicators of everyday memory, with the proviso that this should involve assessing real-world behaviors. We have adopted an everyday prospective memory task widely used in the literature – a laboratory contact task (e.g., Maylor, 1990; Henry et al., 2004; Troyer et al., 2008). Individuals are asked to contact (by telephone, text, or email) our laboratory on five scheduled dates and times, too infrequent to become a habit, but more critically, generating enough trials to allow sensitive measurement of prospective memory errors (Maylor, 1990). We record the number of successful contacts (within a 15-min window of the specified time) and the deviation in time of actual contacts to specified times.

### Empirical Tests of This Intervention Approach

We (Pearman et al., 2020) recently completed our first intervention study using EMMI. The results were highly encouraging. Participants found the intervention accessible, believed it was effective for them, and reported still using the approach in a 1-month follow-up survey. The vast majority of participants were highly enthusiastic about its benefits. Acceptance of the intervention and enthusiasm for its use are a critical aspect of fostering future use of the techniques.

**FIGURE 1:**
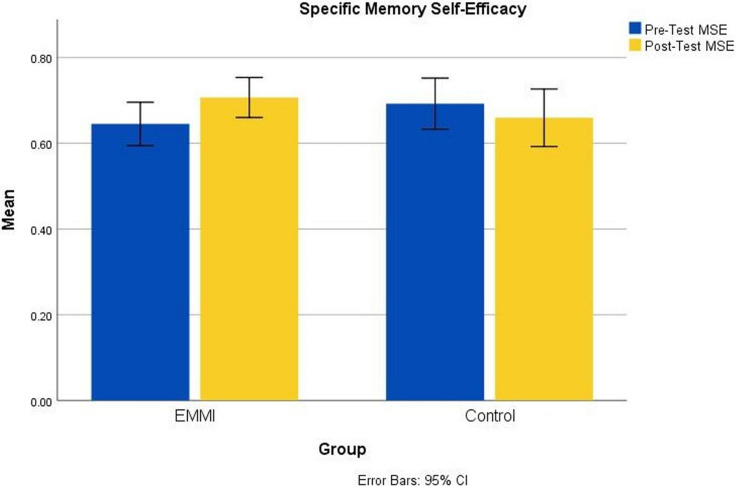
Pretest–Posttest changes in Specific Memory Self-Efficacy. Significant increases were observed in the EMMI group but not in the Control group.

More critically, the major outcome measures demonstrated the intervention’s effectiveness. Memory strategy interventions with an everyday memory component often evaluate changes in subjective memory, such as reduced memory complaints or increases in memory self-efficacy (West et al., 2008; Wiegand et al., 2013). As can be seen in Figure 1, Pearman et al. (2020) detected an increase in an aggregate memory self-efficacy scale (Lineweaver and Hertzog, 1998) in EMMI participants, but no such change in wait-list control participants. The differences in effect sizes were large, *d* = 0.43 for pre-test to post-test change in the EMMI group versus *d* = −0.23 for the control group. This effect was seen on everyday aspects specifically targeted by the intervention, including memory for names, but were not restricted to those aspects. EMMI participants also showed significant increases in perceived control over memory, compared to the controls. This outcome shows that our training program can improve SMCs in older adults by a combined approach of belief restructuring and demonstrated everyday memory effectiveness during training.

**FIGURE 2:**
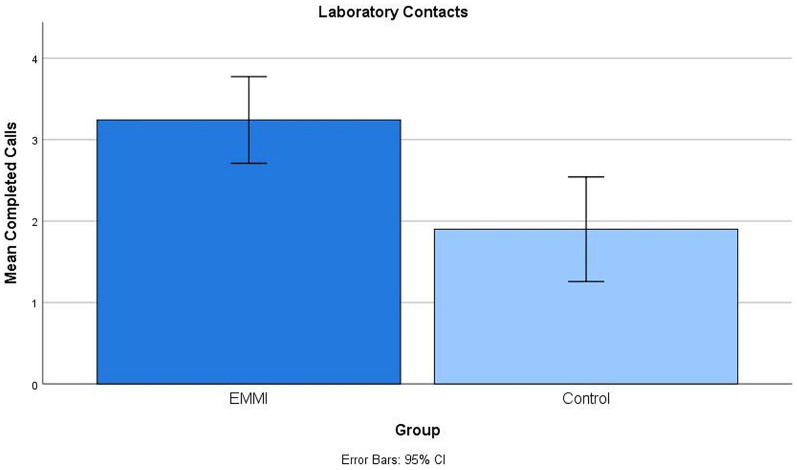
Group differences in completed laboratory contact within a 15-min window of scheduled time. The EMMI group completed significantly more contacts than the Control group.

EMMI participants also manifested much better laboratory contact performance than wait-list controls, completing more of the scheduled lab contacts within a window 15 min before or after the specified contact time (see Figure 2). The effect size was large by conventional standards (*d* = 0.93). Their contact times were also reliably closer to the scheduled time. These outcomes provide an objective performance-based evidence for success of the intervention for everyday prospective memory.

**FIGURE 3:**
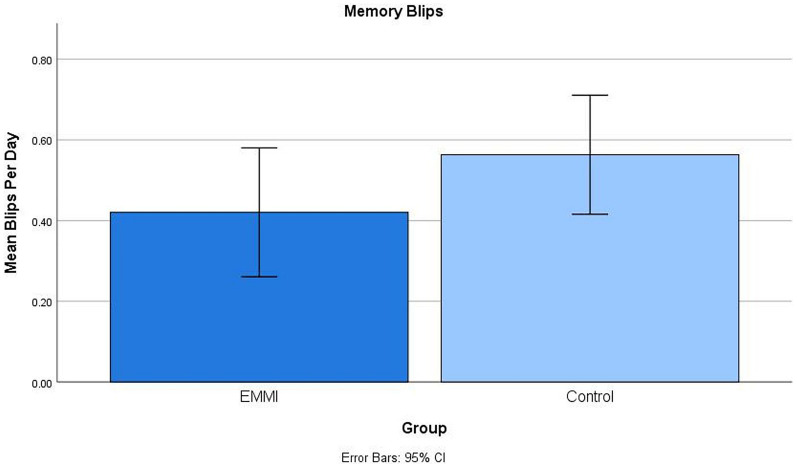
Significant group differences in daily memory successes reported during the assessment window following the shaping period. The EMMI group reported more everyday memory successes than the Control group.

In terms of measures taken from our daily diary, EMMI participants reported more everyday memory successes than controls (*d* = 0.47; see Figure 3) and also reported fewer memory problems during a 10-day assessment window that followed Phase 3 shaping procedures. However, unlike the previously mentioned outcome measures, this effect was not statistically significant after controlling on a set of covariates, including age, education, and gender.

**FIGURE 4:**
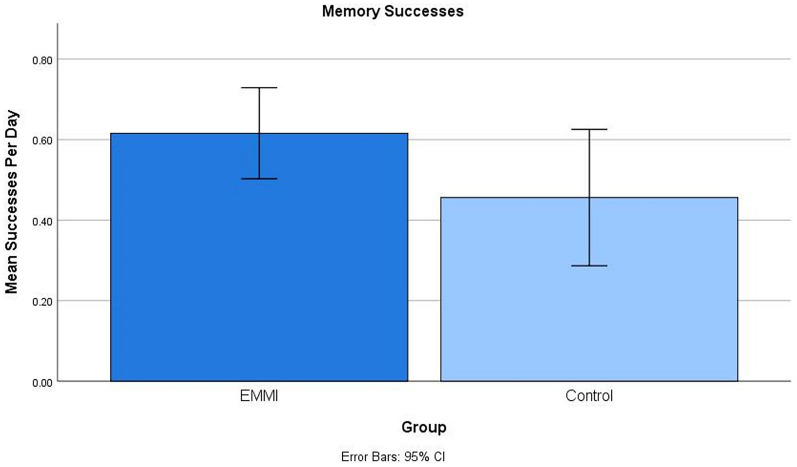
Group differences in daily memory failures (termed “blips”) reported during the assessment window following the shaping period, with the EMMI group reporting fewer everyday memory failures in the daily diaries than the Control group.

They also reported fewer memory errors during the 10-day window (Figure 4), *d* = 0.36, although the difference was not statistically reliable with our sample size, with or without control on covariates.

We regard these outcomes as highly encouraging, in that they provide clear and compelling evidence that our intervention has beneficial impact on everyday memory. Even so, we also identified ways in which the intervention can be improved based on empirical results and feedback from our participants in this first study. These improvements will be incorporated into the next empirical training study that should provide a more definitive test of intervention efficacy.

Pearman et al. (2020) did measure a standardized memory test at pretest and posttest. Because our intervention does not target mnemonic strategy use in testing contexts, we did not expect to see improvements in episodic memory test performance, and none were found. This finding supports the distinction between standard memory test outcomes and everyday memory behaviors, while also demonstrating specific benefits of EMMI to everyday memory.

Our next study will improve our method of collecting self-reports of everyday memory failures. We are currently piloting a new event-based ecological momentary assessment (EMA) procedure that will provide a method for people to rapidly report memory successes and failures in close proximity to the memory incident, in order to avoid some of the retrospective report biases likely present with nightly diaries. We use a smartphone application program that asks individuals to record a short audio clip describing the event as soon as possible after event occurrence. They then have the option of immediately reporting or deferring the report for a brief time if they cannot provide more extensive details about the failure in the moment for any reason. Playback of the audio clip as a rich retrieval cue during diary completion should also help to reduce errors of omission – forgetting everyday memory errors that occurred earlier in the day – when completing the nightly diary (see Schmitter-Edgecombe et al., 2020 for an example of successful use of similar EMA procedures, even with cognitively impaired older adults).

The next study, funded by the National Institute on Aging (NIA R21 AG059942; PI Hertzog) will also conduct a registered randomized controlled trial (RCT#NCT04088136), assigning older adults to participate in either the enhanced EMMI or in a classical memory-strategy intervention with regular telephone contact from research staff following both types of training. We regard this trial as a critical test of our hypothesis that memory strategy interventions, as typically conducted, will improve memory test performance but have minimal impact on everyday memory, as measured by EMA outcomes and performance on the laboratory contact task. Conversely, we expect EMMI to improve everyday memory but have minimal impact on memory test performance, except perhaps through the indirect mechanism of improving confidence in one’s own memory [perhaps avoiding age-related stereotype threat (e.g., Barber, 2020)].

## Conclusion

The intervention approach we review here has a strong chance of meaningfully benefiting everyday memory behavior of older adults. It also ameliorates subjective memory complaints with apparent benefits for life satisfaction and morale. As such, it may promote functional independence and prolong aging in place in individuals experiencing normal age-related cognitive decline. The existing intervention literature also promotes optimism that versions of EMMI could be used in special-needs populations, such as individuals with mild cognitive impairment (Troyer et al., 2008; Kinsella et al., 2018) or Type II diabetes, where self-regulation is challenged by a greater degree of cognitive decline than might be expected from normal aging.

## Data Availability Statement

The original contributions presented in the study are included in the article/supplementary material, further inquiries can be directed to the corresponding author/s.

## Author Contributions

CH wrote the main front end conceptual piece. AP wrote the main part of the description of the intervention protocol. EL and MH were instrumental in providing a descriptive overview of the methodology used in the intervention and generated the figures. All the authors worked collaboratively on the prose refinement of the manuscript.

## Conflict of Interest

The authors declare that the research was conducted in the absence of any commercial or financial relationships that could be construed as a potential conflict of interest.
